# Impact of nutrition since early life on cardiovascular prevention

**DOI:** 10.1186/1824-7288-38-73

**Published:** 2012-12-21

**Authors:** Ornella Guardamagna, Francesca Abello, Paola Cagliero, Lorenzo Lughetti

**Affiliations:** 1 Department of Paediatrics, University of Torino, Piazza Polonia 94, Torino, Italy; 2 Department of Paediatrics, University of Modena & Reggio Emilia, Modena, Italy

**Keywords:** Prenatal nutrition, Breasting, Weaning, Prevention, Cardiovascular diseases

## Abstract

The cardiovascular disease represents the leading cause of morbidity and mortality in Western countries and it is related to the atherosclerotic process. Cardiovascular disease risk factors, such as dyslipidemia, hypertension, insulin resistance, obesity, accelerate the atherosclerotic process which begins in childhood and progresses throughout the life span. The cardiovascular disease risk factor detection and management through prevention delays the atherosclerotic progression towards clinical cardiovascular disease. Dietary habits, from prenatal nutrition, breastfeeding, complementary feeding to childhood and adolescence nutrition play a basic role for this topic.

The metabolic and neuroendocrine environment of the fetus is fundamental in the body’s “metabolic programming”. Further several studies have demonstrated the beneficial effects of breastfeeding on cardiovascular risk factors reduction. Moreover the introduction of complementary foods represents another important step, with particular regard to protein intake. An adequate distribution between macronutrients (lipids, proteins and carbohydrates) is required for correct growth development from infancy throughout adolescence and for prevention of several cardiovascular disease risk determinants in adulthood.

The purpose of this review is to examine the impact of nutrition since early life on disease.

La malattia cardiovascolare rappresenta la principale causa di morbilità e mortalità dei paesi occidentali ed è correlata a degenerazione vascolare aterosclerotica. I fattori di rischio cardiovascolari quali dislipidemia, ipertensione, insulino resistenza e obesità accelerano tale processo il cui esordio è noto sin dell’età pediatrica ed evolve nel corso della vita. L’individuazione e la cura dei fattori di rischio cardiovascolari mediante la prevenzione dei fattori causali ritardano la progressione dell’aterosclerosi e l’insorgenza dei sintomi cardiovascolari. La nutrizione svolge un ruolo preventivo fondamentale sin dall’epoca prenatale e nelle diverse età della crescita.

La condizione metabolica e neuro-endocrino cui è sottoposto il feto è rilevante per la “programmazione metabolica”. E’ dimostrata inoltre l’importanza delle modalità di allattamento e divezzamento con particolare interesse per l’assunzione di proteine nel controllo dei fattori di rischio cardiovascolari. La corretta distribuzione di macronutrienti (lipidi, proteine e carboidrati) dall’infanzia all’adolescenza favorisce una crescita corretta e risulta utile a prevenire l’insorgenza dei determinanti di rischio di malattia cardiovascolare in età adulta.

Nella presente review verrà esaminato l’impatto della nutrizione dalle più precoci fasi delle vita sul rischio cardiovascolare.

## Introduction

Cardiovascular diseases (CVD) represent the leading cause of morbidity and mortality in Western countries. It has been demonstrated that atherosclerosis begins early in life and progresses throughout life span [1,2]. Risk factors, such as dyslipidemia (including high levels of low-density lipoprotein cholesterol [LDL-C], high levels of triglycerides [TG] and low levels of high-density lipoprotein cholesterol [HDL-C]), hypertension, obesity, insulin resistance (IR), accelerate the atherosclerotic process and increase CVD risk. Several studies in children and adolescents have demonstrated that risk factors may be present since childhood, tracking into adult life, so an early identification is necessary to establish a primary prevention approach [3]. Recent evidences from population-based and clinical studies have emphasized the importance of primordial prevention, aimed to avoid risk factors development [3,4].

Several epidemiological studies across a wide range of countries and ethnicities have demonstrated that nutrition, from fetal life ongoing and early human development, exert a profound impact on risk development of adult disease. The theory of the “fetal origins of adult disease” (FOAD) [5-7], as coronary artery disease (CAD) [8], diabetes mellitus (DM) [9], as well as cancer and others pathologies, was formulated. The FOAD hypothesis is based on the premise of “developmental plasticity”: it is the phenomenon by which one genotype can produce different phenotypes in response to different environmental factors. In particular there are specific “critical” periods whereby the organs and systems of the human body are plastic and sensitive to the environment [6,10]. During intrauterine life, nutrition plays an important role for the so called “metabolic programming”: several different stimuli produce permanent changes which persist throughout life span and have long-term implications for later development of metabolic diseases. This programming extends also into early life and childhood [10].

Recent evidences have demonstrated that environmental factors can interact with fetal genome through epigenetic processes, without altering it. Epigenetic mechanisms, comprising DNA methylation and de-methylation, post-transcriptional processes as methylation, phosphorylation and acetylation of core histones, produce heritable changes resulting in phenotype variations [11-13]. These emerging findings can offer opportunities for novel risk biomarkers and for interventions to promote health across the life course [11]. Other mechanisms involved in early life programming are represented by permanent structural changes (eg, the permanent reduction of renal glomeruli or β-cell mass in the endocrine pancreas) and permanent effects on cellular aging regulation (eg, oxidative stress processes) [14].

This review examines the impact of nutrition since early life on CVD risk.

### Prenatal nutrition

Maternal nutritional status influences the quantity and quality of nutrients reaching the fetus, thus representing a potential determinant of metabolic programming and body composition.

Data from several human studies on early-life CVD risk determinants have been primarily focused on birthweight. An inverse association between CVD mortality and birthweight was identified [5,15,16]. Further epidemiological studies have been conducted over the last decades in attempt to determine a possible correlation between birthweight and CVD risk factors. Most of these studies were retrospective and involved adult populations, while few prospective studies were conducted in children. It has been showed that subjects with low birthweight (LBW), especially as a result of intrauterine growth restriction, presented a higher prevalence of arterial hypertension [17-19], IR [19], T2DM [9,20] and metabolic syndrome (MS) [21]. Among birthweight determinants, the mother size and the intrauterine nutrient supply are the most important. In particular intrauterine growth retardation can be induced by prolonged but modest changes in maternal diet or by more severe changes in uterine blood supply near to the pregnancy term.

In relation to nutrients’ quantity, many studies examined the impact of maternal undernutrition on offspring phenotype [22,23] and CVD outcome [24,25]. Hales *et al.* formulated the hypotheses of “thrifty phenotype” which suggested that, when exposed to conditions of deprivation during fetal life, subjects developed metabolic, endocrine and/or anatomical adaptations aimed to immediate survival [26]. The “thrifty phenotype” is characterized by a condition of IR which may be considered as the persistence of fetal response in order to facilitate energy serving and maintain an adequate concentration of glucose for vital organs, particularly the brain. This results in a state of hyperglicemia and increases de novo lipogenesis. The latter is responsible for later disease as IR and diabetes because of the “mismatch” between intra- and extra-uterine environment [27,28]. Further it has been demonstrated that fetal growth restriction alters adipose tissue development. Subjects born small for gestational age have greater body fat percentage than subjects born appropriate for gestational age, and difference in fat distribution track to adulthood [29].

Furthermore another relevant consequence of undernutrition during fetal life is represented by the reduced cell number and growth in many organs, such as kidney. A reduced number of glomeruli, associated with the development of glomerulo-sclerosis, consequent to an increased blood flow over time, favours the development of hypertension in adult life [30].

In the last years a dramatic worldwide increase in overweight and obesity has occurred. This condition of overnutrition had in turn led to a relevant number of pregnant women with gestational obesity and gestational diabetes mellitus thus promoting fetal hyperinsulinemia. These conditions are related to adulthood overweight, obesity, impaired glucose tolerance and MS [31,32].

New evidences from epidemiological and experimental studies have provided data about the impact of maternal diet quality on long-term implications for their offsprings thus supporting the concept of gene–diet interactions modulating CVD risk factors. The modulation by a genetic polymorphism of a dietary component effect on a specific phenotype (e.g., cholesterol levels and obesity), can interact increasing the risk for developing chronic disease. Maternal dietary fat composition is the most important determinant of the quality of fatty acids which are transferred across the placenta [33]. Maternal dietary lipids, including fatty acids, have demonstrated effects on different tissues and metabolic systems: adipose tissue, muscle, liver, pancreas, intestine and hypothalamic appetite/energy sensing pathways. Dietary fatty acids composition can regulate gene expression, inter-cellular communications, metabolic and neuroendocrine networks [34]. Besides the relevance of nutrition itself attention should be paid to the concept of gene–diet interactions and its impact on the clinical outcome. The modulation by a genetic polymorphism of a dietary component effect on a specific phenotype (e.g., cholesterol levels, obesity, hypertension), can interact increasing the risk for developing chronic disease. A body of studies focus on concentrations of specific plasma fatty acids which may interact with genes and are determined by both intake and metabolism. An example is represented by the activity of key enzymes desaturases. The latter are encoded by genes in the FADs cluster that contribute to PUFA concentrations. A genome-wide association study applied to the Italian InCHIANTI population described strong associations for the FADS1, FADS2, and FASD3 variants and PUFA levels. This study identifies sites at which the genetic variability in enzymes metabolizing essential fatty acids interacts with diet thus changing plasma fatty acid concentrations and potentially modulating the genetic risk related to metabolic pathways [35]. Anyway, despite the fact that multiple studies have shown statistically significant interactions between n-3 fatty acids and genetic variants on intermediate and disease phenotypes, the individual level of evidence is very low and recommendations cannot be made on increasing or reducing the intake of n-3 fatty acids based on each individual’s genotype [36]. In the last half-century changes in dietary lipid intake are represented by an excess of ω-6 fatty acids (eg. linoleic acid [LA], arachidonic acid [ARA]), inadequate ω-3 fatty acids (including linolenic acid [ALA], docosahexaenoic acid [DHA] and eicosapentaenoic acid [EPA]) and monounsaturated oleic acid consumption, and high intake of fat from refined triacylglycerols [34]. LA and ALA are mainly derived from vegetable fats and oils while ARA, DHA and EPA are present only in animal tissue lipids, thus meaning that their intake depends mainly on protein dietary sources. Among fatty acids, the role of DHA with regard to brain and retina development is well known [37]. Beside this property, Weisenger *et al.* highlighted the possible effect of DHA on blood pressure (BP) control because they observed that DHA deficiency in the perinatal period resulted in increased BP later in life [38]. So these results suggest that perinatal supplementation of long chain polyunsaturated fatty acids (LCPUFA) could prevent hypertension in adulthood [39].

Heerwagen *et al.* have suggested that maternal-to-fetal fatty acid transfer depends on elevated maternal body and plasma TG concentration producing increased birthweight, body fat composition and elevated inflammatory cytokines [40]. Oxidative stress and inflammation are particularly influenced by *trans* fatty acids derived from partially hydrogenated vegetable oils. These conditions during fetal life predispose to hyperphagia, obesity, IR and diabetes [40].

The effects of protein and carbohydrate intake during pregnancy on offspring long-term health were examined by observational studies, with contrasting results concerning BP [41-44].

Finally it should be mentioned that air and food pollutants may impact on fetal development as demonstrated benzopyrene exposure that may impair fetal growth, particularly in genetically susceptible populations carrying the variant Val allele on the GSTP1 gene [45].

### Breastfeeding

Several studies and statements have demonstrated the potential benefits of breastfeeding on CVD risk reduction.

Human milk is a complex fluid secreted by the mammary gland, containing carbohydrates and salts in solution, casein in colloidal dispersion, cells and cellular debris, and lipids mostly in emulsified globules [46]. In addition human milk contains essential fatty acids, hormones, growth factors, immune-related components, enzymes, polyamines and other biologically active compounds, which may play a relevant role in the health benefits associated with breastfeeding [47].

Concerning human milk lipids (which content is around 50%), they are represented by triacylglycerols (98%), phospholipids (0.8%), cholesterol (0.5%) and many others. The major monounsaturated fatty acid (MUFA), the oleic acid, in addition to the usual role of energy source and structural components, facilitates triacylglycerols metabolism of milk fat globules. Nowadays fatty acids *trans*-isomers represent a hot topic: they originate from the maternal diet, are secreted into human milk and exert damaging effects on infant development, as previous reported by Carlson *et al.*[48].

Exclusive breastfeeding for at least 6 months has been recommended by the European Society of Pediatric Gastroenterology Hepatology and Nutrition (ESPGHAN) because human milk properties are important in promoting optimal growth, neurocognitive development, resistance to infections and cardiovascular health [47,49,50]. Several studies have reported beneficial effects on dyslipidemia, hypertension, reduced glucose tolerance, overweight/obesity prevention.

Data accumulated over the last years have suggested that breastfeeding influences lipid metabolism, determining lower levels of total cholesterol (TC) in adulthood. Owen *et al.* in a systematic review observed higher TC concentrations in breast-fed than in formula-fed infants (<12 months), because of an higher cholesterol content in human milk than in infant formulae (mean TC difference 0.64, 95% CI 0.50 to 0.79 mmol/L), while adulthood TC levels resulted lower in those breast-fed in infancy (mean TC difference −0.18, 95% CI −0.30 to −0.06 mmol/L) [48]. These data were further confirmed by a meta-analysis conducted by the World Health Organization (WHO) [51].

These results suggest a possible effect of breast milk on lipid metabolism by a “nutritional programming” consequent to an early exposure to human milk high cholesterol content. This stimulus may modulate cholesterol metabolism by regulating hydroxymethylglutaril coenzyme A (HMG-CoA) reductase activity or by increasing LDL-receptor activity [52].

The impact of breastfeeding on BP is controversial. In a meta-analysis by Owen *et al.*[53] a slight mean difference in systolic BP (SBP) of −1.10 mmHg (95% CI −1.79 to −0.42) was found in subjects breast-fed while no difference was observed for diastolic BP (DBP) later in life. A subsequent meta-analysis in 2005, including approximately 10.000 subjects from 3 studies, demonstrated a difference in SBP of −1.4 mmHg (95% CI −2.2 to −0.6) and in DBP of −0.5 mmHg (95% CI −0.9 to −0.04) in adults previously breast-fed [54]. These data were confirmed by a meta-analysis conducted by WHO [51]. Martin *et al.* reported that breastfeeding was associated with a lower BP at the age of 7.5 years in children born at term [55], but contrasted with PROBIT study results which did not demonstrate any effect of breastfeeding on BP in children 6.5 years aged [56]. Further a negative association between breastfeeding duration and later BP was observed [55,57].

A possible explanation of the role of human milk in reducing BP is represented by the low sodium content [57,58] associated with the LCPUFA high concentration. LCPUFA are incorporated into cell membranes of the vascular endothelium and increase endothelial nitric oxide synthesis [59]. Further it has been demonstrated that the dietary supplementation with LCPUFA, from birth to 6 months, is associated with a significant reduction in mean BP and DBP at the age of 6 years [60]. Another possible relevant effect can be exerted by human milk lower protein amount compared to infant formulae. The latter higher protein intake could stimulate insulin secretion, thus promoting IR which in turn may increase BP via several mechanisms as stimulation of the sympathetic nervous system, smooth muscle hypertrophy and increased renal sodium retention [61].

A protective role of breastfeeding against glucose intolerance and T2DM development has been demonstrated in several studies. Owen *et al.* highlighted, in a review including results from 7 studies, the possible preventive effect of breastfeeding on T2DM development (OR 0.61, 95% CI 0.41-0.85). These results evidenced lower blood glucose and serum insulin levels in infancy and lower insulin levels later in life [62]. Similar results were also reported in the WHO meta-analysis [51]. These effects can be attributed to human milk components, in particular to hormones such as leptin, adiponectin, resistin and ghrelin [63]. Considering leptin, it plays a fundamental role in glucose metabolism regulation since early life. This hormone, particularly, improves insulin sensitivity by reducing intracellular lipid levels in liver, skeletal muscle and pancreatic β-cells [64]. Also adiponectin contributes to regulate glucose homeostasis: it increases insulin action in the liver as well as glucose metabolism in the skeletal muscle [65]. It should be remarked that adiponectin correlates with neonatal growth parameters and adiposity. Further resistin shows a role in maintaining metabolic neonatal homeostasis and it may exert little or no contribution to IR [66]. Considering ghrelin, there are contrasting data about its influence on insulin secretion [63].

Breastfeeding could play a key role in preventing overweight/obesity across the life course as reported by numerous systematic reviews and reports [67-71]. But how breastfeeding exert these effects? Breastfeeding potential benefits on body composition may be due to the slower pattern of growth, in particular related to the weight gain, in breastfed compared with formula-fed infants [72], so the former show a lower risk of obesity. These outcome difference could be explained by human and formula milk composition and by infants’ nutritional behaviour.

About formula and human milk composition, the latter has a lower protein and a slightly lower energy content. It has been demonstrated that a high protein intake in infancy enhances the secretion of insulin and insulin growth factor 1 (IGF-1) thus stimulating cell proliferation, accelerating growth and increasing adipose tissue deposition, with an early occurrence of adiposity rebound [73]. These evidences have been confirmed in a randomized clinical trial, by Koletzko *et al.*, who found a positive association between infant formula protein content and higher weight gain in the first 2 years of life [74]. Further in recent years it has been better understood the role of breast milk hormones on obesity prevention: leptin, adiponectin, IGF-1, ghrelin, obestatin and resistin regulate energy balance and food intake and modulate neuroendocrine mechanism involved in body weight regulation [75].

From a behavioural point of view a possible explanation could be the self regulation achieved by breast-fed babies that persist later in life [47].

### Introduction of complementary foods

Recommendations by ESPGHAN Committee on Nutrition have suggested that exclusive breastfeeding should be pursued for around 6 months to achieve optimal growth, development and health [47] and complementary foods could be introduced between 17 and 26 weeks [76]. At this time human milk would be insufficient to satisfy energy, protein, some fat-soluble vitamins and micronutrients requirements. Among weaning timing determinants, it has been observed that infant weight plays an important role as heavier infants tended to be weaned earlier than age-matched lighter infants [77]. About the relationship between new food introduction and infant growth, Wilson *et al.* demonstrated a higher prevalence of obese children at 7 years of age among those who were given solid food before 12 weeks of age [57].

Several studies evaluated the relationship between protein intake from complementary foods and obesity risk. Some data have demonstrated that a protein intake of 4–5 g/kg wt per day (corresponding to 16-20% of total energy intake) at the age of 8–24 months is associated to an overweight higher risk later in life [78]. This association was not observed when a dietary protein intake was limited to 3.75 g/kg wt per day (corresponding to 15% of total energy intake) [73]. The association between high-protein intake and early adiposity rebound has been explained by Agostoni *et al.* and Ketelslegers *et al.*: dietary protein could stimulate the activity of IGF-1, determining both adipocyte differentiation and adipogenesis [73,79]. About the relationship between protein intake during the complementary period and BP, no definitive conclusions are available at present. Infancy may be a greater salt sensitive period than later life [80] and it has been demonstrated that an excess of dietary sodium intake in newborns and young infants could raise BP [81] while a salt restriction during the first year of life significantly slows down the BP rise in childhood [82]. Furthermore the effect of LCPUFA intake during the complementary feeding period on later BP was considered by a trial in which 9-month-old infants were randomized to receive a fish oil supplement for 3 months or no supplement [83]. At 12 months a lower SBP (−6 mmHg) was reported in those children who received fish oil supplementation. These results are in accordance with the follow-up study by Forsyth *et al.*[60].

Concerning the fat intake during the weaning period, several studies did not found any association with subsequent weight or fatness in infants and children [84,85]. In the DONALD Study Karaolis *et al.* demonstrated that, among normal growers, a high fat intake at both 12 and 18–24 months resulted in a normal decrease in body fat composition between 2 and 5 years, on the contrary to rapid growers [84]. Further a significant association between fat intake and subsequent weight and body fat mass was observed after 2 years of age. So these evidences suggest that fat intake should not be restricted during the first 2 years of life, except for rare lipid disorders which require fat restriction since early infancy. The ESPGHAN Committee on Nutrition has recommended that fat content should provide at least 25% of the total energy intake in order to guarantee a correct growth and neurocognitive development [76]. It has been demonstrated that overconsumption of energy-dense complementary foods could induce excessive weight gain in infancy, with an higher risk of obesity in childhood and adulthood [86,87].So the quantity and the quality of foods given during the weaning period are fundamental, in particular as feeding practices during the first year of life provide the basis for food habits in childhood and impact on later health in adulthood.

### Nutrition in childhood and adolescence

Nutrition in childhood and adolescence may play a relevant role in the development of CVD in adulthood. The National Cholesterol Education Program Pediatric Panel Report provided dietary recommendations for all children as part of a population-based approach to reduce CVD risk for the first time in 1992 [88]. These recommendations were revised in 1998 [89] and 2008 [90]. In addition in 2011 the Expert Panel on Integrated Guidelines for Cardiovascular Health and Risk Reduction in Children and Adolescents published a Summary Report which provided evidences regarding the efficacy of specific dietary intervention to reduce CVD risk [3].

It has been suggested that the total fat intake should be limited to 30% of daily calories, saturated fat intake limited to 7-10% of calories and dietary cholesterol limited to 200–300 mg/day. The remaining fat intake should derived from MUFA (10%) and polyunsaturated fatty acids (PUFA) (10%); further *trans* fatty acids should be avoided. Protein and carbohydrate intakes should account for 15-20% and 50-55% of daily calories, respectively. These dietary indications, concerning in particular daily fat intake, are recommended for children older than 2 years [88].

In the prospective Special Turku Coronary Risk Factor Intervention Project (STRIP), over than 1.000 infants 7 months aged, recruited from the general population, were randomized to receive a low-saturated-fat, low-cholesterol diet (total fat of 30-35% of calories, saturated fat/MUFA+PUFA ratio of 1:2 and cholesterol intake < 200 mg/day) or an unrestricted diet [91]. These subjects were followed up during childhood and adolescence, and parameters including dietary intakes, serum lipids, BP, somatic growth and sexual maturation were monitored. It was demonstrated that intervention boy group had lower cholesterol values than controls between 13 and 60 months of age, while a slightly but no significant difference was found in females. TG concentration instead was similar in the 2 groups [92]. The difference in TC levels was further confirmed in boys at the age of 7 years, together with slightly larger LDL particles in the intervention group [93], and persisted through at least the first 14 years of life [94]. The effect on LDL particles should be underlined because large LDL are demonstrated to be less atherogenic than small dense LDL. Another significant observation was represented by a lower prevalence of obesity in girls in the intervention group, compared with controls [95].

In the STRIP study follow-up Niinikoski *et al.* reported the positive effect of the restriction of saturated fat from infancy until 15 years of age on BP. SBP and DBP resulted 1.0 mm Hg lower (95% CI for systolic: -1.7 to −0.2; 95% CI for diastolic: -1.5 to −0.4) in children receiving low-saturated-fat counselling than in control children [96]. The explanation of these BP variations in the intervention group can be partly attributed to the quality of fats assumed, rather than the quantity, as saturated fat intake can determine central obesity, related to insulin sensitivity. The latter resulted improved during the intervention period, thus contributing to lower BP [97]. Other variables such as a higher intake of PUFA, a higher protein intake, fibers amount, or a combination of all of these, can exert a significant effect on BP. Overall it should be underlined that STRIP supervised dietary regimen reduces the exposure to cardiovascular risks without any adverse effects on growth, cognitive and pubertal development.

The relationship between BP and dietary macronutrients in children have been estimated by other studies, with contrasting findings. Boulton J. observed that greater intakes of energy, fats, and carbohydrates were associated with lower BP in 2 years old children while no any association with protein intake was detected [98]. On the contrary, Ulbak *et al.* did not found any association between BP and intakes of fats, saturated fatty acids, PUFA, while a negative association between MUFA intake (daily energy percentage and g per day) and SBP was reported. This study also demonstrated that protein intake, expressed as daily energy percentage and as g per day, was significantly associated with lower SBP and DBP in 2.5 years old children [99]. Concerning gender, Jenner DA *et al.* observed in 9 years old Australian children that SBP was negatively associated to calorie-adjusted protein intake in girls, while no detectable relationships between BP and calorie-adjusted intakes of fats, carbohydrates, sodium, potassium, calcium, magnesium was reported [100]. The possible effect of protein on BP could be explained by the vasodilating properties of certain amino acids, which determine an increased production of nitric oxide [101]. Fiber intake might also influence children’s BP [99,102].

It has been reported by many studies that BP tracks from early childhood into adulthood [103]. Recent data from the Cardiovascular Risk in Young Finns Study have been published about the relationship between childhood diet, with particular regard to fatty acids quality, and BP in adulthood in more than 800 individuals who participate in the 27 years follow-up. Kaikkonen *et al.* have highlighted the positive independent association between childhood serum cholesterol ester fatty acid (CEFA) proportion and SBP and DBP in adolescence and adulthood [104]. These results were stronger in men than in women and stronger for SBP than for DBP. In particular fatty acids derived from animal fats of dietary origin seemed to be linked with higher BP in adulthood, while fatty acids derived from vegetable oils and margarine appeared to be associated with lower BP. Looking at fatty acids fractions, in men the proportions of saturated fatty acids, MUFA and ω-3 PUFA were positively associated with BP while ω-6 PUFA were negatively associated with BP. In women weaker associations between CEFA proportions and BP were reported.

A dietary intervention is mandatory when CVD risk factors are present since childhood. The Dietary Intervention Study in Children (DISC) evaluated the effect of a restricted saturated fat and cholesterol diet in a cohort of 663 children affected by elevated LDL-C levels, aged 8 to 11 years, with a mean of 7.5 years follow up [105]. It was found that the dietary intervention could improve lipid profile over 3 years with intensive adherence, although not significantly over 5 years, probably as a consequence of a lower-intensity maintenance intervention. In the DISC study the relationship between dietary nutrients and BP was also investigated [106]. Controlling for all nutrients, total fats were significantly directly associated with both SBP and DBP, and fiber was significantly inversely associated with DBP. So these results provide evidence of the potential role of dietary fats, and possibly fiber, on BP levels in children. Further studies are required to provide conclusive findings.

Other dietary measures useful for CVD prevention such as to encourage high dietary fiber intake from food, to avoid sugar-sweetened beverage intake, and to limit sodium intake were suggested by the Expert Panel [3]. Dietary fiber is an important constituent of diet: it promotes gastrointestinal function and helps to prevent CVD risk factors by lowering serum total and LDL-C [107] and serum insulin concentrations and by reducing the risk of obesity as demonstrated in young adults [108]. Dietary fiber intake is inversely associated with energy density and with increased levels of body fat and is positively associated with nutrient density. A daily dietary fiber intake of at least age plus 5 g for young children up to 14 g/1.000 kcal for older children and adolescents is recommended.

Several evidences have demonstrated that high added sugar consumption, in particular in the form of sugar-sweetened beverages, is associated with cardiovascular risk factors, both independently and through the development of obesity [109,110].

A healthy dietary model widely recognized is represented by the Mediterranean diet. The beneficial effects of this food intake regimen on CVD and other major chronic diseases have been demonstrated by several studies in different populations, in epidemiological, population based and randomised clinical trials [111-113]. The traditional components of this dietary pattern are represented by whole cereals (complex carbohydrates), vegetables and fruits (fiber, vitamins, antioxidants), nuts (PUFA), legumes (vegetal proteins), fish (proteins and PUFA) and virgin olive oil (MUFA and antioxidants). Serra-Majem *et al.* demonstrated in a cohort of more than 3.000 Spanish subjects, aged 6–24 years, the high nutritional quality of the Mediterranean diet, particularly in children and adolescents [114]. In a recent meta-analysis by Nordmann *et al.* considering overweight/obese adults with at least one additional cardiovascular risk factor, the Mediterranean diet appeared to be more effective than low-fat diets in inducing clinically relevant long-term changes in CVD risk factors and inflammatory markers [115]. Thus this dietary pattern should be promoted since childhood and adolescence in order to prevent CVD later in life.

## Conclusions

This review highlights several evidences from epidemiological, population based and randomized clinical trials taking in account the nutrition impact on CVD burden.

Maternal nutritional status represents a potential determinant of metabolic programming and body composition affecting fetal life ongoing. This programming extends also into infancy and childhood: the quantity of energy intake and particularly the quality of macronutrients influence growth and CVD risk factor development.

Finally this relevant body of knowledge should be considered in the clinical practice, since prenatal life, in order to develop preventive and intervention strategies to promote health across life course, aimed to CVD prevention.

## Abbreviations

ALA: Linolenic acid; ARA: Arachidonic acid; BP: Blood pressure; CAD: Coronary artery disease; CEFA: Cholesterol ester fatty acid; CVD: Cardiovascular diseases; DHA: Docosahexaenoic acid; DISC: Dietary interventio dietary intervention study in children; DBP: Diastolic BP; ESPGHAN: European society of pediatric gastroenterology hepatology and nutrition; EPA: Eicosapentaenoic acid; FOAD: Fetal origins of adult disease; HDL-C: High-density lipoprotein cholesterol; HMG-CoA: Hydroxymethylglutaril coenzyme A; IGF-1: Insulin growth factor 1; IR: Insulin resistance; LA: Linoleic acid; LBW: Low birthweight; LCPUFA: Long chain polyunsaturated fatty acids; LDL-C: Low-density lipoprotein cholesterol; MUFA: Monounsaturated fatty acid; MS: Metabolic syndrome; PUFA: Polyunsaturated fatty acids; SBP: Systolic BP; STRIP: Special turku coronary risk factor intervention project; TC: Total cholesterol; T2DM: Type 2 diabetes mellitus; TG: Triglycerides; WHO: World health organization.

## Competing interests

The authors declare that they have no competing interests.

## Authors’ contributions

All the authors contributed to the review and were involved in writing, revising and approving the final draft of the manuscript.
